# Consensus Recommendations for the Diagnosis and Management of X-Linked Hypophosphatemia in Belgium

**DOI:** 10.3389/fendo.2021.641543

**Published:** 2021-03-19

**Authors:** Michaël R. Laurent, Jean De Schepper, Dominique Trouet, Nathalie Godefroid, Emese Boros, Claudine Heinrichs, Bert Bravenboer, Brigitte Velkeniers, Johan Lammens, Pol Harvengt, Etienne Cavalier, Jean-François Kaux, Jacques Lombet, Kathleen De Waele, Charlotte Verroken, Koenraad van Hoeck, Geert R. Mortier, Elena Levtchenko, Johan Vande Walle

**Affiliations:** ^1^ Centre for Metabolic Bone Diseases, University Hospitals Leuven, Leuven, Belgium; ^2^ Division of Pediatric Endocrinology, KidZ Health Castle, University Hospital Brussels, Vrije Universiteit Brussel (VUB), Brussels, Belgium; ^3^ Department of Pediatric Endocrinology, University Hospital Ghent, Ghent, Belgium; ^4^ Department of Pediatric Nephrology, Antwerp University Hospital, Antwerp, Belgium; ^5^ Laboratory of Experimental Medicine and Pediatrics, University of Antwerp, Antwerp, Belgium; ^6^ Pediatric Nephrology, Cliniques Universitaires St. Luc (UCL), Brussels, Belgium; ^7^ Paediatric Endocrinology Unit, Hôpital Universitaire des Enfants Reine Fabiola, Université Libre de Bruxelles, Brussels, Belgium; ^8^ Department of Endocrinology, University Hospital Brussels, Vrije Universiteit Brussel (VUB), Brussels, Belgium; ^9^ Department of Orthopaedic Surgery and Department of Development and Regeneration, Prometheus LRD Division of Skeletal Tissue Engineering, KU Leuven - University Hospitals Leuven, Leuven, Belgium; ^10^ XLH Belgium, Belgian X-Linked Hypophosphatemic Rickets (XLH) Patient Association, Waterloo, Belgium; ^11^ Department of Clinical Chemistry, University Hospital Center of Liège, University of Liège, Liège, Belgium; ^12^ Physical Medicine, Rehabilitation and Sports Traumatology, University and University Hospital of Liège, Liège, Belgium; ^13^ Division of Nephrology, Department of Pediatrics, University Hospital Center of Liège, Liège, Belgium; ^14^ Unit for Osteoporosis and Metabolic Bone Diseases, Department of Endocrinology and Metabolism, Ghent University Hospital, Ghent, Belgium; ^15^ Department of Medical Genetics, Antwerp University Hospital and University of Antwerp, Antwerp, Belgium; ^16^ Department of Pediatrics/Pediatric Nephrology, University Hospitals Leuven, Leuven, Belgium; ^17^ Department of Pediatric Nephrology, University Hospital Ghent, Ghent, Belgium

**Keywords:** burosumab, fibroblast growth factor 23 (FGF23), osteomalacia, rickets, vitamin D, X-linked hypophosphatemia

## Abstract

X-linked hypophosphatemia (XLH) is the most common genetic form of hypophosphatemic rickets and osteomalacia. In this disease, mutations in the *PHEX* gene lead to elevated levels of the hormone fibroblast growth factor 23 (FGF23), resulting in renal phosphate wasting and impaired skeletal and dental mineralization. Recently, international guidelines for the diagnosis and treatment of this condition have been published. However, more specific recommendations are needed to provide guidance at the national level, considering resource availability and health economic aspects. A national multidisciplinary group of Belgian experts convened to discuss translation of international best available evidence into locally feasible consensus recommendations. Patients with XLH may present to a wide array of primary, secondary and tertiary care physicians, among whom awareness of the disease should be raised. XLH has a very broad differential-diagnosis for which clinical features, biochemical and genetic testing in centers of expertise are recommended. Optimal care requires a multidisciplinary approach, guided by an expert in metabolic bone diseases and involving (according to the individual patient’s needs) pediatric and adult medical specialties and paramedical caregivers, including but not limited to general practitioners, dentists, radiologists and orthopedic surgeons. In children with severe or refractory symptoms, FGF23 inhibition using burosumab may provide superior outcomes compared to conventional medical therapy with phosphate supplements and active vitamin D analogues. Burosumab has also demonstrated promising results in adults on certain clinical outcomes such as pseudofractures. In summary, this work outlines recommendations for clinicians and policymakers, with a vision for improving the diagnostic and therapeutic landscape for XLH patients in Belgium.

## Introduction

X-linked hypophosphatemia (XLH) is the most common genetic form of hypophosphatemic rickets and osteomalacia. Its incidence has been estimated at 3.9-5 cases per 100.000 live births, with no evidence of ethnic variation (1, 2). Belgium has an annual birth rate of ± 117.800 (year 2018), ± 1.93 million growing children (< 15 years) and 11.4 million total population (2019 census) (3). Extrapolation of the ~1:20.000 incidence and prevalence would imply that this rare disease has an incidence of less than 6 cases in newborns annually and a prevalence of 97 and 475 cases in the pediatric and adolescent/adult population, respectively. Other sources have reported prevalences lower than 1:20.000 *e.g.* 1.4 per 100.000 in the United Kingdom (4) to 1.7 per 100.000 in Norway (5). Possible reasons include gaps in diagnosis and referral of XLH patients from primary or secondary care to centers of expertise. This is certainly the case also in Belgium, where only recently efforts have been initiated to improve the care for patients suffering from rare/orphan diseases (6).

The pathophysiology of XLH has been reviewed extensively elsewhere (7, 8). In brief, mono-allelic mutations or chromosomal derangements affecting the Phosphate Regulating Endopeptidase Homolog, X-Linked (*PHEX*) gene on the X chromosome lead to elevated levels of the hormone fibroblast growth factor 23 (FGF23), resulting in renal phosphate wasting, impaired 1α-hydroxylation of 25-hydroxyvitamin D [25(OH)D] to the active hormone calcitriol (1,25-dihydroxyvitamin D [1,25(OH)_2_D]) and consequently, chronic hypophosphatemia, impaired skeletal mineralization and rickets (9). In children, the corresponding clinical features may include delayed growth and short stature, craniosynostosis and raised intracranial pressure, deformities of weight-bearing limbs, muscle weakness, gait abnormalities (10), tooth abscesses and excessive dental caries (9, 11, 12).

Following growth plate closure, a part of adolescent and young adult patients continue to experience debilitating symptoms while others may experience a “honeymoon” phase (similar to other metabolic bone diseases) with fewer musculoskeletal problems (except dental manifestations). During this phase, conventional therapy with phosphate supplements and active vitamin D analogues is often stopped, because subjective and skeletal benefits are thought to be lacking (13). During adolescence, the psychological burden increases (14), which may contribute to poor adherence and lack of follow-up. The historic perception of therapeutic futility in adults has probably contributed to the dearth of transitional care programs between pediatric and adult specialty care for XLH patients. Nevertheless, even adults with milder forms usually develop symptoms in their third or fourth decade, which may include bone and joint pain, fatigue, enthesopathy (commonly involving the hips and anterior spinal ligament), pseudofractures, dental complications and early osteoarthritis (15). These complications ultimately cause chronic pain, impaired mobility, loss of productivity and lower quality of life (9, 15–20). Extraskeletal complications include hearing loss, symptomatic Chiari malformations, arterial hypertension [(possibly induced by oral phosphate supplements (21, 22)]. An increased prevalence of overweight and obesity has also been observed in XLH (23). Recent data suggest that XLH may be associated with increased risk of mortality in older adults, but not in children (4). A recent population-based study also reported an increased risk of depression and socioeconomic deprivation (24).

Recently, international evidence-based guidelines for the diagnosis and management of XLH have been published (13, 25). However, efforts are also required to translate the principles outlined in these guidelines to more practical recommendations at the national level, considering local elements such available resources and health economic aspects (26). Towards this aim, and as part of an interdisciplinary effort to improve the diagnostic and therapeutic care pathway for XLH patients in Belgium, a multi-stakeholder panel gathered to develop national consensus recommendations.

## Methods

First, two in-person meetings were held between several of the authors (ML, JS, NG, EB, CH, JLa, KH, EL and JV) to review the available evidence, facilitate discussion and to propose diagnostic and therapeutic criteria for Belgium. The international evidence-based guidelines (13, 25) as well as recent randomized trials were considered as the basis for practical recommendations applicable to the Belgian context. Further input was sought from all other co-authors through consecutive e-mail rounds. The writing panel is composed of national experts from all universities and involves specialists in pediatric nephrology, endocrinology, adult metabolic bone diseases, rheumatology, clinical genetics, orthopedic surgery, clinical chemistry and physical medicine and rehabilitation. Furthermore, a Belgian XLH patient who is a founding member of the Belgian patient organization (XLH Belgium) as well as of its French counterpart (RVRH-XLH) participated. This work was co-authored by members of and endorsed by the Belgian Society for Pediatric Nephrology, the Belgian Society for Pediatric Endocrinology and Diabetes, the Flemish Network on Rare Bone Diseases, the Belgian Bone Club, the Royal Belgian Society of Laboratory Medicine and the Royal Belgian Society of Physical and Rehabilitation Medicine.

## Results

### Diagnosis

The diagnosis of XLH relies on the combination of clinical, radiographic, biochemical and genetic features (25). More specifically, this involves signs of rickets and/or osteomalacia in association with hypophosphatemia and renal phosphate wasting in the absence of vitamin D deficiency. The diagnosis should be confirmed by genetic testing whenever possible.

The clinical features include those common for hypophosphatemic rickets, as outlined in the introduction *i.e.* short stature, waddling gait, and leg bowing in growing children, in addition to muscle weakness. Fatigue and chronic pain become more prevalent in older children and particularly adults. Growth delay usually becomes evident from 9-12 months of age in XLH children (27). Early diagnosis and treatment is associated with better outcomes in children. Even when plasma phosphate is measured, hypophosphatemia may be overlooked due to lack of attention, misinterpretation of reference values in children, or waxing and waning of phosphatemia. In adults, signs of prior rickets during childhood should be sought *e.g.* short stature and limb bowing, although these may be absent in patients with milder phenotypes or those having received appropriate treatment during childhood. Some clinical features distinctive for this form of hypophosphatemic rickets are dental abscesses and enthesopathy, which may present to rheumatologists and are sometimes mistaken for spondylarthropathies.

Hypophosphatemic rickets has a wide differential diagnosis (**Table 1**). Although XLH is the most common genetic form, both acquired and rarer inherited differential-diagnoses should be considered. Neither clinical, biochemical, radiographic or genetic examinations on their own can correctly distinguish XLH from other conditions. Therefore, we recommend a multimodal work-up of suspected XLH by an experienced clinician to exclude other diseases. Bone biopsy is not routinely recommended in XLH (13). Moreover, expertise in bone histomorphometry is still scarcely available in Belgium (mainly in collaboration with neighboring countries, although bone histomorphometry recently became reimbursed through the national health insurance).

**Table 1:** Differential diagnoses of X-linked hypophosphatemia (XLH).

Disease (gene)	Biochemical	Radiographic	Clinical
**XLH (*PHEX*)**	FGF23↑, 1,25(OH)_2_D↓, (Ca↓), PTH↑, calciuria↓	Dense bones, BMD↑, pseudofractures, enthesopathy	Rickets + **dental abscesses, enthesopathy**
**Vitamin D-deficiency rickets (nutritional, *CYP2R1, CYP3A4*)**	**25(OH)D↓,** PTH↑, (Ca↓), (1,25(OH)_2_D↓), calciuria↓, **FGF23↓**	**Osteopenia, fractures**	Rickets (symptomatic **hypocalcemia**)
**VDRR1A (*CYP27B1*)**	1,25(OH)2D↓, (Ca↓), PTH↑, calciuria↓	**Osteopenia, fractures**	Rickets (symptomatic **hypocalcemia**)
**Chronic renal insufficiency**	FGF23↑, $PO_{4}^{3-}$ **↑,** 1,25(OH)_2_D↓, (Ca↓), PTH↑, calciuria↓	**Unremarkable**, hyperparathyroidism	**No rickets** (unless nutritional)
**Fanconi syndromes, renal tubular acidosis**	FGF23↑, 1,25(OH)_2_D↓, (Ca↓), PTH↑, calciuria↓ **+ metabolic acidosis, low urate, glucosuria, amino-aciduria, some GFR↓**	Rickets and/or osteomalacia	**Evidence of underlying disorders**
**Tumor-induced rickets/osteomalacia**	(FGF23↑), 1,25(OH)_2_D↓, (Ca↓), PTH↑, calciuria↓	Rickets and/or osteomalacia	**No family history of rickets or osteomalacia**
**HHRH, NPHLOP1/2 (*SLC34A3, SLC34A1, SLC9A3R1*)**	FGF23↑, **1,25(OH)_2_D↑,** (Ca=↑), **PTH↓, calciuria↑**	**Osteopenia, fractures**	Rickets and/or osteoporosis, **prominent nephrocalcinosis/nephrolithiasis**
**Jansen metaphyseal chrondrodysplasia**	FGF23↑, **1,25(OH)_2_D↑,** (Ca=↑), **PTH↓, calciuria↑**	**Osteopenia, fractures**	**Very short stature, more pronounced skeletal dysplasia**
**ADHR (*FGF23*)**	**Variably FGF23↑ associated with iron deficiency**		May be milder, no rickets in adult-onset forms
**ARHR1 (*DMP1*)**	FGF23↑, 1,25(OH)_2_D↓, (Ca↓), PTH↑, calciuria↓	**Dense vertebral bodies**	**May present as sclerosing bone disease**
**ARHR2 (*ENPP1*)**	FGF23↑, 1,25(OH)_2_D↓, (Ca↓), PTH↑, calciuria↓	**Generalized arterial calcifications**	**Generalized arterial calcifications ± multisystem manifestations**
**ARHR3, Raine syndrome (*FAM20C*)**	FGF23↑, 1,25(OH)_2_D↓, (Ca↓), PTH↑, calciuria↓	Dense bones, BMD↑, pseudofractures, enthesopathy	**Cerebral calcifications; perilacunar osteomalacia on bone biopsy; facial features**
**α-klotho (*KL* translocation)**	FGF23↑, **α-klotho↑,** (1,25(OH)_2_D↓), (Ca↓), PTH↑, calciuria↓	Rickets	**Macrocephaly, prominent frontal bossing, and dysplasia of the nasal bones, with exaggerated midfacial protrusion**
**FD/MAS, linear sebaceous nevus syndrome (post-zygotic somatic mutations)**	FGF23↑, 1,25(OH)_2_D↓, (Ca↓), PTH↑, calciuria↓	**Focal bone lesions**	**Café-au-lait spots or nevi; focal bone lesions, jaw involvement**
**Osteoglophonic dysplasia (*FGFR1*), opsismodysplasia (*INPPL1*)**	FGF23↑, 1,25(OH)_2_D↓, (Ca↓), PTH↑, calciuria↓	**Severe bone dysplasias; non-ossifying bone lesions, hypo-/adontia**	**Very short stature; severe skeletal dysplasia**
***(SGK3)***	Unclear pattern	Rickets	Rickets

Bold, distinctive biochemical, radiographic, or clinical features allowing distinction from XLH. BMD, bone mineral density.

#### Biochemical Work-Up and Differential Diagnosis

**Figure 1:**
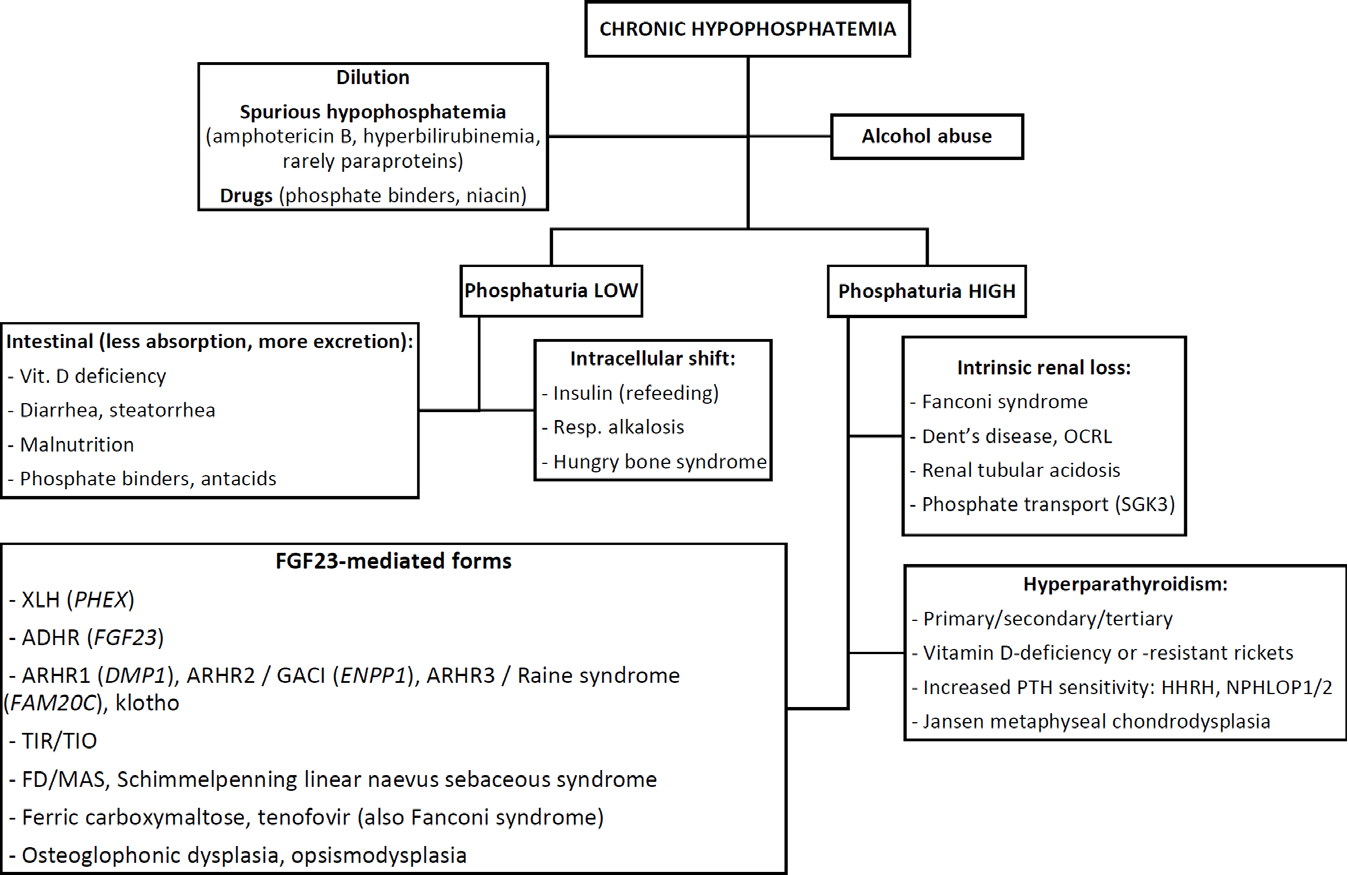
Flowchart outlining the differential-diagnosis of chronic hypophosphatemia based on biochemical features. See text for abbreviations.

**Figure 1** shows a practical flowchart outlining the differential-diagnosis of hypophosphatemia in children or adults, according to biochemical features. The clinical, radiographic, biochemical and pathophysiological or genetic features of these causes are discussed in detail in this section.

As with the approach to any electrolyte disorder, the first step after careful history taking and clinical examination is to exclude obvious causes such as dilution (*e.g.* by massive fluid resuscitation, dialysis, plasmapheresis), spurious hypophosphatemia (from *e.g.* drug interference like amphotericin B, interference by bilirubin (28) or specific paraproteins), medication effects [*e.g.* excessive phosphate binders, niacin (29)] or alcohol abuse. Hypophosphatemia in alcoholics has a complex, multifactorial and incompletely understood pathophysiology. These causes should be considered first, since they can usually be diagnosed without further work-up.

##### Distinguishing Acquired vs. Genetic and Acute vs. Chronic Hypophosphatemia

Previously normal plasma phosphate levels suggest three possibilities: an acquired chronic cause, an acquired acute causes or a genetic, adult-onset cause. However, prior phosphate levels are often unavailable. Elevated alkaline phosphatase (ALP) is also indicative of chronic hypophosphatemia and consequent rickets/osteomalacia. Hypophosphatemia in the absence of rickets should raise suspicion for either an acute, transient cause (*e.g.* intracellular shift from hyperventilation, refeeding, hungry bone syndrome) or an acquired chronic cause such as alcohol abuse, tumor-induced rickets/osteomalacia (TIR/TIO) or certain medications such as tenofovir or frequent ferric carboxymaltose infusions (30). Notably, some genetic forms of hypophosphatemia may have an adult onset (notably, autosomal-dominant hypophosphatemic rickets, see below), in which case signs of rickets may be absent.

Chronic hypophosphatemia is believed to play a central role in the pathogenesis of almost all forms of rickets (31, 32). After confirming chronic hypophosphatemia, the next step is to assess phosphaturia *i.e.* whether hypophosphatemia is due to renal phosphate wasting or not (see *Biochemical Work-Up: Pre-Analytic and Analytic Considerations* below).

Once renal phosphate wasting has been confirmed, three mechanisms of renal phosphate loss remain: (i) defective intrinsic renal phosphate transport, (ii) parathyroid hormone (PTH)-mediated (and/or vitamin D-mediated) hyperphosphaturia, or (iii) FGF23-mediated causes.

##### Defective Intrinsic Renal Phosphate Reabsorption

The first category includes all causes of Fanconi syndrome *i.e.* a more generalized low molecular weight solute wasting at the proximal tubulus level. Low levels of uric acid and bicarbonate, glucosuria, aminoaciduria and low molecular weight (tubular) proteinuria should hint towards this category. At an older age, there may be slowly progressive renal insufficiency. However, some degree of renal tubular acidosis may be acquired during long-standing XLH, particularly when associated with nephrocalcinosis (33). The differential-diagnosis of Fanconi syndrome itself is broad and includes (non-exhaustively) mitochondrial respiratory chain diseases, metal intoxications, Wilson’s disease, cystinosis, multiple myeloma [especially when involving light chains (34)], Sjögren’s disease, medications like ifosfamide or tenofovir, genetics forms of renal tubular acidose, Dent’s disease and Lowe’s oculocerebrorenal syndrome (from *OCRL1* or *CLCN5* mutations).

Some genetic conditions may rather selectively impair renal phosphate reabsorption, including several undiagnosed diseases. Recently, mutations in *SGK3* (serum/glucocorticoid-regulated kinase 3) were identified as a possible cause of autosomal-dominant hypophosphatemic rickets (35), although further study is needed to understand the underlying mechanisms.

##### Parathyroid Hormone-Mediated Hyperphosphaturia

The second category entails disorders of increased PTH or impaired vitamin D actions. Recent evidence shows elevated FGF23 in primary hyperparathyroidism (36–38). Both XLH and primary hyperparathyroidism may feature hypophosphatemia, renal stone formation, elevated PTH and FGF23 levels. Still, primary hyperparathyroidism should be easily distinguished by the presence of hypercalcemia, hypercalciuria, and high-normal to elevated 1,25(OH)_2_D as well as by the absence of rickety features.

Occasionally, it may be difficult to distinguish patients with non-familial XLH (*i.e.* probands without other affected family members) from children with nutritional, vitamin D-deficient- or -resistant rickets. Low 25(OH)D levels are common in XLH patients and should be corrected before the diagnosis is confirmed. In patients refractory to 25(OH)D replenishment, supervised supplementation may distinguish between poor compliance *vs*. resistance due to inactivating *CYP2R1* (recessive) or activating *CYP3A4* (autosomal-dominant) mutations (39).

Because FGF23 stimulates *CYP24A1* and inhibits *CYP27B1* (25-hydroxyvitamin D_3_-1α-hydroxylase) expression in both renal and extrarenal tissues (40), FGF23-mediated forms of hypophosphatemia are associated with low 1,25(OH)_2_D levels (41), secondary hyperparathyroidism (42), and low-normal to slightly decreased calcemia. However, 10-16.7% of adult XLH patients may exhibit tertiary (hypercalcemic) hyperparathyroidism, which is amenable to parathyroidectomy (42, 43). The levels of 1,25(OH)_2_D are also decreased in patients with very severe 25(OH)D deficiency, those with chronic renal insufficiency or in rare cases of *CYP27B1* mutations (vitamin D-resistant rickets type 1A). In the latter three situations, FGF23 levels are low, high or unknown, respectively. Vitamin D-resistant rickets due to inactivating vitamin D receptor mutations is exquisitely rare and more easily distinguished from XLH due to elevated 1,25(OH)_2_D levels and because almost all patients display alopecia. Importantly, radiographic features prominently differ between XLH and nutritional or vitamin D-related forms of rickets, with dense bone cortices in XLH and radiolucent bones in vitamin D-related rickets. Thus, X-ray imaging as well as genetic testing may distinguish these disorders (see **Table 1**).

Hereditary hypophospatemic rickets with hypercalciuria (HHRH) is a group of disorders featuring increased renal sensitivity to PTH. Bi-allelic mutations in *SLC34A3* cause HHRH, while heterozygous mutations in *SLC34A1* and *SLC9A3R1* cause the phenotypically similar hypophosphatemic nephrolithiasis/osteoporosis syndromes (NPHLOP1 and NPHLOP2, respectively). In all these conditions, patients may have hypophosphatemia, elevated FGF23 levels and nephrolithiasis/nephrocalcinosis (7). However, osteopenia/low bone mineral density, hypercalciuria, high-normal to elevated 1,25(OH)_2_D and low PTH point to the diagnosis of HHRH/NPHLOP. The same common and distinctive features apply to the ultrarare skeletal dysplasia Jansen metaphyseal chondrodysplasia, which is caused by activating PTH receptor 1 mutations (44). Additional clinical features in Jansen metaphyseal chondrodysplasia patients include severe short stature, brachycephaly, micrognathia, hypertelorism, and clinodactyly.

##### Fibroblast Growth Factor 23-Mediated Renal Phosphate Wasting

The third pathophysiological category involves FGF23-mediated forms of hypophosphatemic rickets. After XLH, the second most common genetic form of hypophosphatemic rickets is autosomal-dominant hypophosphatemic rickets (ADHR). Notably, it may be difficult to distinguish autosomal- and X-linked dominant inheritance in certain families. ADHR is caused by specific mutations in the *FGF23* gene (mostly involving amino acid residues R176 and R179), making the intact (biologically active) hormone resistant to cleavage (45). Since iron deficiency increases *FGF23* expression, this gene-environment interaction explains why adult-onset ADHR may be unmasked by iron deficiency and may be cured with (oral) iron supplements alone (46–50). Symptoms may wax and wane in parallel with iron loss during *e.g.* menstruation or pregnancy. As noted above, adults with ADHR may not have signs of rickets.

Ferric carboxymaltose (Injectafer^®^) infusions are a common drug-induced cause of transient (and sometimes symptomatic) hypophosphatemia, mostly in patients without chronic renal insufficiency. While the clinical significance in patients requiring sporadic infusions remains unknown, frequent infusions are a potential cause of acquired hypophosphatemic rickets and/or osteomalacia. The underlying mechanism involves specific carbohydrate moieties in the carboxymaltose apomolecule, which interfere with FGF23 cleavage. This explains why other i.v. iron formulations do not cause hypophosphatemia, and why intact (active) FGF23 increases more than c-terminal FGF23 in ferric carboxymaltose-induced hypophosphatemia (30, 51, 52). Interestingly, FGF23 may also be increased in tenofovir-induced Fanconi syndrome (53), *via* yet unknown mechanisms.

Autosomal-recessive forms of hypophosphatemic rickets (ARHR) may be caused by mutations in dentin matrix protein 1 (*DMP1*, as in ARHR1) or ectonucleotide pyrophosphatase/phosphodiesterase 1 (*ENPP1*, as in ARHR2). ARHR1 may present as a sclerosing bone dysplasia with hyperostosis and very dense vertebral bodies (54). *DMP1* as well as *PHEX* are highly expressed in osteocytes, although it remains unknown how they alter the phosphate-FGF23 set point (55). ENPP1 is a critical enzyme in the generation of the mineralization inhibitor pyrophosphate. Loss-of-function mutations in *ENPP1* lead to generalized arterial calcification of infancy (GACI). Many children with *ENPP1* mutations who survive GACI will go on to develop ARHR2 (56, 57). The mechanism underlying raised FGF23 (58) and hypophosphatemia in GACI/ARHR2 remains incompletely understood. Extraskeletal features of GACI including hearing loss (59), thrombocytopenia, neurologic, cardiovascular, hepatic manifestations and hypoglycemia have recently been described (60). Recently, a case of ARHR was described with compound mutations in *DMP1* and *SPP1* (encoding osteopontin, another small integrin-binding ligand, N-linked glycoprotein [SIBLING] protein family member) (61).

FGF23 effects on the kidney are mediated by its co-receptor klotho. One case report described a balanced translocation affecting the *KL* gene, resulting in elevated α–klotho levels and hypophosphatemic rickets with elevated FGF23 (62).

FGF23 is believed to be cleaved at specific sites mainly by FURIN (PCSK3) (63), which also cleaves PTH and several other substrates. This cleavage is further regulated *via* phosphorylation by FAM20C (family with sequence similarity 20, member C, also known as DMP4; the gene mutated in Raine syndrome) and O-glycosylation by GALNT3 (polypeptide N-acetylgalactosaminyltransferase 3) (63–65). Raine syndrome (also called type 3 ARHR) was formerly believed to be a lethal osteosclerotic bone dysplasia, although now survivors into adulthood have been recognized (66–68). Clinical, biochemical and radiographic features of Raine syndrome may be very similar to sporadic XLH, but distinctive facial features, cerebral calcifications and osteomalacia surrounding the osteocyte lacunae on bone biopsy are a typical hallmark of this condition (69). In contrast, recessive, loss-of-function *GALNT3* mutations impair FGF23 actions and thus, like in genetic or autoimmune FGF23 deficiency, lead to familial hyperphosphatemic tumoral calcinosis syndrome (70, 71).

In non-familial childhood- or adult-onset cases of hypophosphatemia, one must always bear in mind the possibility of tumor-induced hypophosphatemic rickets (TIR, in growing children) or TIO (72). In TIR and TIO, small mesenchymal tumors secrete FGF23 and/or other phosphatonins such as matrix extracellular phosphoglycoprotein (MEPE) (73). Thus, circulating FGF23 is usually but not always raised in TIR/TIO. Typical clinical features include chronic and progressive bone pain, muscle weakness and low bone mineral density. Diagnosis of TIO and TIR often remains extremely difficult, leading to extensive (and sometimes unnecessary) diagnostic procedures, since tumors may be too small for detection by conventional radiological methods (see *Imaging Studies* below).

Hypophosphatemic rickets in several sclerosing bone, skin or soft tissue disorders is believed to result from increased secretion of FGF23 or related phosphaturic hormones by the aberrant cells (74). Such may be the case in fibrous dysplasia/McCune-Albright syndrome (FD/MAS, which are caused by post-zygotic, somatic *GNAS* mutations) and Schimmelpenning-Feuerstein-Mims syndrome (which both feature bone lesions and café-au-lait spots), as well as in isolated linear nevus sebaceous syndrome (the latter two caused by post-zygotic somatic *KRAS*, *HRAS* or *NRAS* mutations). Finally, FGF23-mediated hypophosphatemia has been reported in two ultrarare skeletal dysplasias: osteoglophonic dysplasia (characterized by rhizomelic dwarfism, craniosynostosis, impacted teeth, hypodontia or anodontia, and multiple non-ossifying bone lesions) (75) and opsismodysplasia (a rare spondylo(epi)chondrodysplasia characterized by delayed skeletal maturation) (76).

#### Biochemical Work-Up: Pre-Analytic and Analytic Considerations

The measurement of plasma calcium (preferably ionized if feasible, or alternatively albumin-corrected), phosphate, ALP, creatinine, PTH and 25(OH)D are part of the standard work-up for any form of rickets or osteomalacia (77).

##### Alkaline Phosphatase

In the absence of liver disease, bone-specific ALP comprises ~90% of total ALP in children but only ~50% in adults (78, 79). Therefore, bone-specific ALP has been recommended for the monitoring of XLH in adults (25). Although bone-specific ALP is available in Belgium, it is still rarely used. In children, age-specific reference values should be used for correct interpretation of these analyses (particularly for ALP and phosphate). During the first months of life, plasma phosphate and ALP may be normal in XLH. ALP has no significant diurnal variation and can be used to monitor disease activity (taking age-specific changes during growth into account), as well as adherence to therapy (see *Monitoring* below).

##### Plasma and Urinary Phosphate

Since plasma phosphate fluctuates and is influenced by dietary intake, the international gold standard to assess phosphaturia is to determine the maximal tubular reabsorption of phosphorus per glomerular filtration rate (TmP/GFR), ideally from a fasted, second morning paired plasma and urine phosphate and creatinine sample (or 2-hour fasted morning urine collection) (80). Age-related reference ranges for TmP/GFR have been published (81). Coincidentally, the lower limit of normal for phosphate and TmP/GFR in children are numerically similar (82). The fractional tubular resorption of phosphate (TRP) may be within normal limits in children or adults with XLH. On the other hand, hypophosphatemia with a TmP/GFR < 0.85 points to a hyperphosphaturic mechanism. Other proposed definitions of hyperphosphaturia include 24h phosphaturia > 100 mg or hypophosphatemia with a fractional excretion of phosphate > 5%.

A 24h urine collection (or split 22h – 2h collection) may be useful to identify hypercalciuria, which may point to other diagnoses or excessive use of active vitamin D or calcium supplements. There are various definitions of hypercalciuria, either based on total excretion (> 200 to 250 mg/24h or 5.0 to 6.2 mmol/24h in adult women, > 250-300 mg/24h or 6.2 to 7.5 mg/24h in adult men (83), or > 4 mg/kg [0.1 mmol/kg] body weight/day in children) or based on urinary calcium concentration (> 200 mg/L) (84). Clearly, further work is needed to define optimal calciuria cut-points in different populations. The calcium/creatinine ratio allows to adjust for over- or undercollection in 24h urine collections, and may be a more practical (although less sensitive) method to allow the use of spot urine samples in children (85, 86). The addition of hydrochloric acid to the collection ensures dissolution of calcium crystals, thus preventing underestimation of calciuria (87).

##### 1,25-Dihydroxyvitamin D

While the interpretation of calciuria and phosphaturia rely mostly on correct sample collection and interpretation of reference values, 1,25(OH)_2_D and FGF23 are more challenging laboratory analyses. Levels of 1,25(OH)_2_D are higher during childhood, and pediatric reference ranges have recently been proposed (88). International efforts to harmonize measurement of 1,25(OH)_2_D as well as PTH are underway (89).

##### Fibroblast Growth Factor 23

Like PTH, FGF23 is an unstable protein susceptible to decay in several fragments. Similar to phosphate, FGF23 shows diurnal variation; early-morning venous samples are recommended, while fasting appears to have little influence on FGF23 (90). Several FGF23 immunoassays are available: four methods for intact FGF23 (from Immutopics, Kainos, DiaSorin and Millipore) and one for c-terminal FGF23 (from Immutopics, which measures both intact molecules and c-terminal fragments). These methods differ not only in their marked use (only DiaSorin is marked for use in diagnostics), cost and compatibility with several automated laboratory platforms, but also in their reference ranges and even in their units of measurement (91, 92). First-generation assays required collection in protease inhibitor-coated tubes, but storage of samples on ice and immediate transport to the lab for prompt centrifugation is nowadays sufficient (92, 93). C-terminal but not intact FGF23 concentrations are much lower in serum than in EDTA plasma samples (93, 94). In treated patients with an unclear diagnosis, it is recommended to stop phosphate supplements (for at least two weeks) before measuring FGF23, because phosphate supplements may increase FGF23. Burosumab therapy (see *Burosumab* below) may cause analytical interference with certain FGF23 assays (95), but FGF23 measurements are not recommended during the follow-up of XLH patients (see *Burosumab* below). Thus, standardization and harmonization of FGF23 assays remain lacking, and results should always be interpreted cautiously. Recently, within- and between-subject biological variability for FGF23 have been published (96). Reference ranges are not universally established: while c-terminal FGF23 concentrations may be higher in children than in adults, they are rather similar for intact FGF23 (82). In Belgium, measurements of FGF23 and 1,25(OH)_2_D are reimbursed only once per year when requested by an internal medicine or pediatric specialist to evaluate abnormal calcemia or phosphatemia.

#### Imaging Studies

A skeletal survey using conventional radiography is useful in the work-up of XLH, to confirm rickets or distinguish it from other skeletal dysplasias (see **Table 1**), and to look for complications such as pseudofractures. X-rays of the knees or wrists are usually sufficient to confirm rickets in children. In adults, X-rays typically show enthesopathy, early spinal and extra-spinal osteoarthritis and/or pseudofractures (which often go clinically and biochemically undetected). However, radiation exposure limits the use of radiography during follow-up, particularly in children. In that regard, low-dose biplanar full-leg X-ray imaging using the EOS^®^ system may be useful (97). In children, radiological signs of rickets in the hand, knees and lower limbs include long bone deformities and abnormal growth plates with widened and frayed metaphyses. Plain radiographs can confirm suspected rickets or can be useful pre-operatively, while clinical and biochemical evaluation (rather than routine repeated X-rays) is more important during follow-up.

Rickets of any cause can be graded using the Rickets Severity Scale (RSS), which has been validated in XLH (98). Higher RSS values (indicating more severe rickets) are associated with more severely impaired growth, walking ability, pain and physical disability (98), making this not only a radiographic but also a clinically relevant outcome. However, this score requires significant expertise [as evidenced by its moderate intra- and inter-rater reliability (98)] and is not yet widely available in Belgium.

Renal ultrasound can be used without radiation harm, to investigate the presence and/or severity of nephrocalcinosis and nephrolithiasis, although this requires an experienced operator. Panoramic dental X-rays may be required for stomatological work-up. Magnetic resonance imaging of the skull base may be indicated when there is concern for Chiari malformations (*e.g.*, persistent headache, neurological or respiratory abnormalities) or to identify calcifications in suspected Raine syndrome (X-ray computed tomography may also be used in adults for the latter purpose). Bone scintigraphy may show increased metaphyseal uptake (99) in all forms of rickets and osteomalacia, which may be mistaken for other conditions *e.g.* avascular necrosis, transient migratory osteoporosis, *etc*. Routine technetium bone scans are not recommended in XLH and only useful to identify focal bone dysplasias such as FD/MAS.

Patients with XLH and several other sclerosing hypophosphatemic diseases usually have elevated bone mineral density Z- or T-scores on dual-energy X-ray absorptiometry (DXA). However, this can usually be appreciated well enough from available plain X-rays. Although low Z- or T-scores may point to other diagnoses such as HHRH/NPHLOP or TIR/TIO, DXA is not recommended in the work-up nor in the follow-up of XLH. Bone ultrasound and high-resolution quantitative computed tomography have also shown increased cortical and trabecular bone mass in XLH patients, although these techniques are still considered investigational (100) and not clinically useful.

Imaging plays a central role in the work-up of TIR/TIO, in order to determine whether the tumor can be surgically resected or requires alternative non-operative treatment. These tumors are often small and elusive, but they commonly arise in the lower limbs or head and neck area and may be located by whole-body magnetic resonance imaging, octreotide single photon emission computed tomography, positron emission tomography with ^18^fluorodeoxyglucose or DOTATOC/DOTATATE tracer, and/or by systemic venous sampling (101–103).

#### Genetic Testing

When the clinical, biochemical and radiographic findings suggest the diagnosis of XLH or another genetic disorder, genetic testing is recommended, both to confirm the diagnosis as well as for genetic counselling of the patient and his/her family members.

As the protean example of an X-linked dominant disorder, XLH is inherited from father to daughter and from mother to children of either sex. There is no convincing evidence that males (who are X-chromosome hemizygous) are more severely affected clinically or biochemically (13, 17, 104–107),. However, there may be a tendency that, compared to girls and women, affected boys and men may display more growth delay (5) as well as greater dysmorphic features such as larger head circumference, greater cranial height, shorter limbs and greater trunk length (108–110).

Genetic testing by Sanger sequencing or next-generation sequencing is readily available in several genetic centers in Belgium for *PHEX* as well as for other skeletal dysplasia genes using a whole-exome sequencing-based gene panel. Genetic testing also examines the 3’ untranslated region in which mutations associated with milder XLH phenotypes have been reported (111–113). In general however, mutations can affect any exon, without a clear genotype-phenotype correlation (114).

Up to 90% of patients clinically diagnosed with XLH will show a *PHEX* mutation (115–117). False-negative testing may occur in case of somatic mosaicism, large deletions, intronic and/or splice site mutations (118, 119). Quantitative polymerase chain reaction or multiplex ligation-dependent probe amplification may be useful to identify such cases (120). Lower diagnostic rates ~50% are obtained when applying *PHEX* sequencing to any unexplained hypophosphatemic rickets (121). Still, because it is the most common genetic form, single-gene *PHEX* sequencing may be the appropriate first step in FGF23-mediated hypophosphatemic rickets possibly due to XLH (122).

Genetic counselling is recommended before obtaining genetic testing, and afterwards to explain the results and implications. Especially in young adults and those planning pregnancy, counselling of the patient and his/her partner is warranted. If the underlying genetic defect is known, patients have a choice between natural conception or preimplantation genetic diagnosis (18). A genetic diagnosis in offspring can be performed, usually soon after birth.

A practical summary of the recommendations for the clinical, biochemical, radiographic and genetic work-up for suspected XLH in Belgium is shown in **Table 2**. These recommendations are generally consistent with the recent international guidelines (25).

**Table 2:** Recommended diagnostic and monitoring tests for XLH in Belgium.

**Clinical**	History (current illness, review of systems, medications, alcohol use, sleep disturbances, mobility)*^†^ Clinical examination including: • height and growth velocity, signs or rickets (limb bowing, chest, …), intermalleolar and intercondylar distance*^†^ • dysmorphic features, head circumference and shape, craniosynostosis, signs of intracranial hypertension (fundoscopy if possibly symptomatic)*^†^ • weight and blood pressure (particularly in patients receiving phosphate supplements)*^†^ • dental examination, mobility, motor development and muscle function (6MWT)*^†^ • bone tenderness, joint range of motion, spine examination, entheses*^†^ • hearing assessment*^†^ • skin (naevi, café-au-lait spots)
**Biochemical**	Recommended tests: • plasma calcium (ionized or albumin-adjusted)*^†^, phosphate^†^, (bone-specific) ALP*^†^, creatinine*^†^ • PTH^†^, 25(OH)vitamin D*^†^, 1,25(OH)_2_D^‡^ • 24h calciuria (or spot urine calcium/creatinine ratio)^†^ For differential-diagnostic purposes: • TmP/GFR (preferably from early morning fasted urinary and plasma creatinine and phosphate) • Bicarbonate, uric acid, glucosuria, amino-aciduria, low molecular mass proteinuria Optional (interpret with caution): • FGF23, intact or c-terminal
**Radiological**	Recommended tests: • Lower extremity and wrist X-ray (including bone age): baseline + repeat when clinically indicated, considering radiation exposure (consider skeletal survey in adults, low-dose biplanar X-ray imaging) • Renal ultrasound (baseline + repeat every 1-2 years during follow-up)*^†^ Not recommended in XLH: • Bone densitometry (DXA)
**Genetic**	Recommended for diagnosis: • Genetic counselling • *PHEX* single gene testing • If negative or other genetic cause more likely: multi-gene panel

*Recommended for monitoring in patients not receiving medical therapy, every 3–6 months (children) to every 6–12 months (adults).

^†^Recommended for monitoring and dose adjustments in patients receiving medical therapy, every 1–3 months (children) to every 3–6 months (adults) (more frequent follow-up may be recommended during the start-up phase of medical therapy).

^‡^Recommended for safety monitoring every 3–6 months in patients receiving burosumab therapy.

### Multidisciplinary Care and Follow-Up

The recent international guidelines suggest follow-up of XLH patients by multidisciplinary teams (25). Individualized goals should be determined. Follow-up should focus on patient-centered outcomes and improving quality of life, by detecting and addressing complications early and monitoring compliance with treatment. Attention should be paid to the impact of the disease on other family members too.

We recommend that these multidisciplinary teams are organized by an expert in metabolic bone diseases and involve both pediatric and adult specialists, nurses, physiotherapists, social workers, psychologists, dietitians and occupational therapists (123). In Belgium, organization of multidisciplinary care can be facilitated by means of conventions *e.g.* for children with chronic kidney diseases or for metabolic diseases. The need for a specific convention for patients (children and adults) with a metabolic bone disease is stressed to finance these multi-disciplinary teams. Currently, children with XLH are mostly followed by pediatric endocrinologist and pediatric nephrologists, whereas adults are mostly followed by metabolic bone disease specialists and endocrinologists in Belgium.

**Figure 2:**
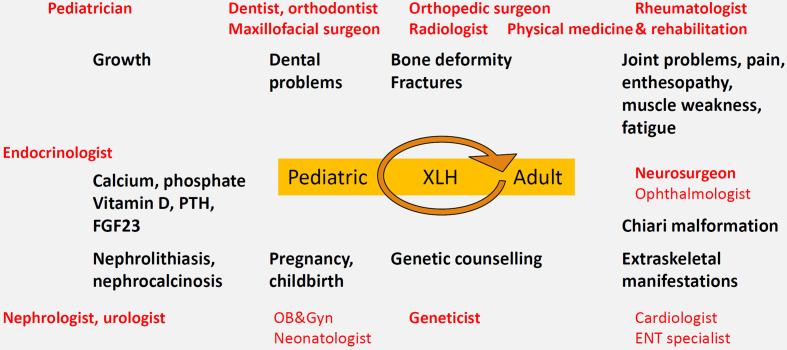
Multidisciplinary care model centered on the XLH patient, with attention to local protocols and transition from pediatric to adult metabolic bone specialist care.

We recommend the development of local protocols for transitional care between pediatric and adult metabolic bone disease specialists, as well as family-based outpatient clinics with pediatric-adult collaboration. In our experience, affected parents sometimes feel inappropriately guilty and/or neglect their own health to focus on their affected children (124). Several specialties should be available on a systematic or consulting basis including dentists, orthodontists and maxillofacial surgeons, pediatric and adult endocrinologists, nephrologists, rheumatologists, orthopedic surgeons, neurosurgeons, radiologists, geneticists, physical medicine and rehabilitation specialists, urologists, otolaryngolostist, ophthalmologists (to perform fundoscopy) *etc*. (**Figure 2**). Of note, since metabolic bone diseases is not a recognized separate specialty in Belgium, the lead specialist may differ by hospital. General practitioners in primary care play an important role in primary recognition of the disease and general follow-up *e.g.* with regards to compliance, extraskeletal manifestations and co-morbidities such as arterial hypertension or obesity, pain, side effects of treatment and psychosocial issues.

The follow-up interval should be individualized to the patient, with more regular follow-up in young or growing children (on average every 3 months) compared to adults (who may be followed every 6 to 12 months, depending on their treatment, symptoms and needs) (25). XLH patients should see their dentist at least twice yearly. These intervals are however based on expert opinion (25).

Clinical assessment including height, weight, inter-malleolar and intercondylar distances, oral/dental and musculoskeletal examination and blood pressure measurement should be performed at every visit in children (see **Table 2**). Biochemical and/or radiological investigations should be evaluated only when clinically indicated.

In asymptomatic adult patients not receiving medical therapy, there is little need for repeated biochemical or radiological testing more than once a year. However, because vitamin D deficiency is common in Belgium and even more common in XLH patients, we recommend monitoring of 25(OH)D at least every twelve months, especially during the winter time, regardless of whether the patient receives medical treatment or not. ALP measurements are a useful indicator of skeletal complications such as progressive rickets/osteomalacia and/or pseudofractures, and therefore should be considered for the monitoring of patients not receiving medical therapy. In asymptomatic untreated patients, monitoring phosphate, 1,25(OH)_2_D or FGF23 levels is not useful.

### Treatment

#### Non-Pharmacological Measures

The overarching goals of treatment should be patient-centered and focus on optimizing quality of life, mobility, pain and minimizing school and work absenteeism. The need for early treatment is stressed in children as it leads to better outcomes, such as improved linear growth, fewer bone deformities and better dental health. Patient education is essential at every visit, particularly in rare diseases and in adolescents. We encourage caregivers to provide patients with information about the nascent XLH patient association in Belgium. Multidisciplinary care should involve physiotherapists as well as psychological, nutritional and social support. In children, it might be necessary to liaise with school physicians to exchange information about *e.g.* growth and hearing problems, to ensure psychosocial wellbeing, and to ensure that medication or therapist care can be provided during school hours when necessary.

A healthy diet with sufficient nutritional calcium intake from dairy products is recommended in XLH, in contrast to calcium supplements, which are relatively contraindicated due to increased risk of kidney stone formation (25). Smoking should strongly be discouraged. Given the increased prevalence of arterial hypertension, overweight, obesity and metabolic syndrome in XLH, weight management and cardiovascular prevention should be an integral part of care. Notably, specialists should maintain a low index of suspicion for both obstructive as well as central sleep apnea in at-risk subjects *e.g.* those with Chiari malformations, those who are overweight or obese, and those with arterial hypertension, fatigue or sleep complaints.

Physical exercise has a myriad of health benefits and is strongly recommended although no formal guidance in XLH exists. Recommendations analogous to those in ankylosing spondylitis and osteoarthritis can be considered (125–127). Children and adults often display limited range of motion in the lower limbs and spine, with consequent gait abnormalities (128). We recommend that exercises and physiotherapy should aim to prevent or improve muscle weakness, back and joint pain, stiffness and limited mobility, by targeting muscle strength, core stability, joint range and general mobility, *e.g.* by resistance exercise training in combination with swimming, yoga, Pilates, dancing *etc*. Participation in leisure or professional sport activities is encouraged, with an emphasis on sports with lower risk of trauma. Targeted rehabilitation is often necessary following surgery and in case of enthesopathy or osteoarthritis. Given that XLH is a rare metabolic and growth disorder causing significant structural and functional musculoskeletal impairments, patients may be eligible for increased reimbursement of physiotherapy [“E-pathology list”, §L (129)]. In case of particularly disabling symptoms and functional impairment, multidisciplinary rehabilitation could be offered in general hospitals or rehabilitation centers under the supervision of a specialist in physical medicine and rehabilitation.

#### Analgesia

Chronic musculoskeletal pain is common in XLH adults and may be caused intrinsically by hypophosphatemia and osteomalacia/rickets (bone and muscle pain) and/or aggravated by (pseudo)fractures, enthesopathy, osteoarthritis *etc*. Based on history and clinical examinations, targeted imaging (using conventional X-ray and ultrasound imaging, MRI or scintigraphy if necessary) should always be considered to allow medical and/or surgical management of the underlying cause. Paracetamol has limited if any benefit, but may be considered for acute pain. Local and/or systemic non-steroidal anti-inflammatory drugs (NSAIDs; with gastroprotection if necessary) in combination with physical therapy (aiming for a good balance between exercise and rest, warmth or ice application, *etc*.) are considered first-line therapies. Although NSAIDs have been theorized to reduce phosphaturia, a randomized trial in children showed no improvement (130). Tramadol and strong opioids may be required for more advanced, otherwise untreatable musculoskeletal problems. Glucocorticoids or colchicine are not recommended. Given uncertainty regarding benefit and safety for calcific enthesopathy in general and lack of data in XLH, we recommend against the use of shockwave lithotripsy.

#### Phosphate and Active Vitamin D Analogs

**Figure 3:**
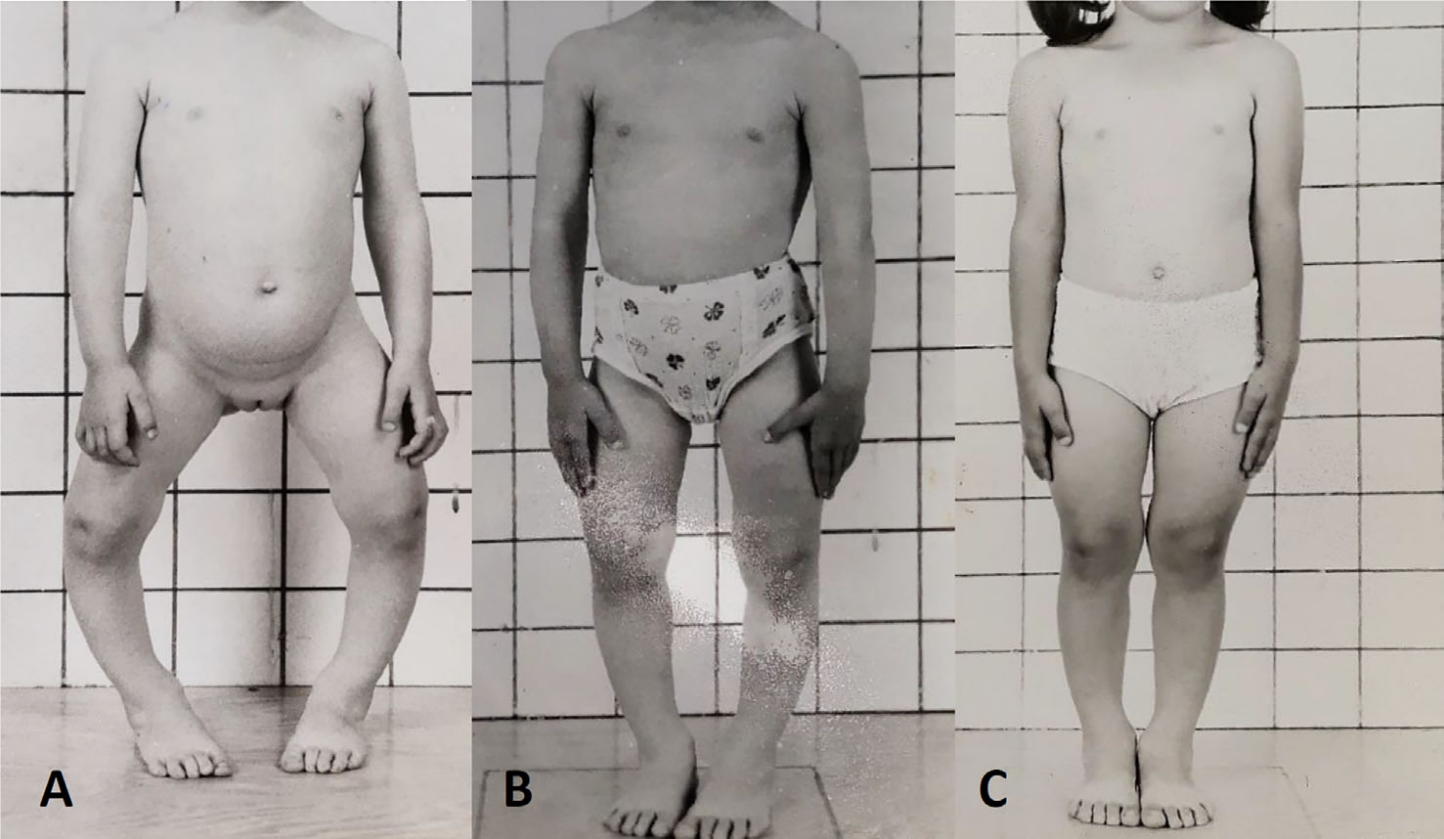
Response to conventional medical treatment with phosphate and active vitamin D supplements in XLH. **(A)** Clinical presentation at age 3 years. **(B)** One year, **(C)** 2 years after initiation of conventional therapy.

The goal of medical treatment in children is to improve rickets, reduce skeletal deformities and avoid the need for surgery, improve height velocity and reduce bone pain (**Figure 3**). Early treatment in children is associated with improved outcomes (131). Although evidence was lacking previously, recent data suggest that conventional therapy may also reduce the burden of severe dental complications (132–135).

Conventional therapy in XLH consists of combination therapy with phosphate and active vitamin D analogs (preferably alfacalcidol, which has a wider therapeutic range and longer half-life than calcitriol and is available in liquid form for children). Phosphate supplements should not be prescribed without vitamin D analogues, since phosphate alone promotes secondary hyperparathyroidism and thereby renal phosphate wasting.

Conventional therapy is burdensome, since it requires multiple daily oral doses. Phosphate supplements are expensive, not reimbursed and generally cumbersome to obtain in Belgium. Financial provisions are available however *via* so-called “conventions” for tubulopathies in children or metabolic diseases in adults. Alfacalcidol is cheap, reimbursed in Belgium upon annual request by a specialist, but supply has occasionally been interrupted by stock breaches. Phosphate salts may be given in effervescent tablets imported from abroad, or in magistral capsules. In young children phosphate salts are usually given in several liquid formulations, such as galenic solutions (Joulie potio or others), i.v. phosphate solutions given orally, or commercial solutions. The choice of the formulation should be based on patient preferences and not solely to the habits of the prescriber. Side effects of oral phosphate include diarrhea, abdominal pain, bloating and secondary hyperparathyroidism. Spreading the dose throughout the day may alleviate side effects. Some patients prefer to dissolve phosphate in a bottle of drinking water, which facilitates spreading intake over the entire day and may reduce side effects. Furthermore, the high sodium and/or potassium load of phosphate supplements may predispose to arterial hypertension and/or hyperkaliemia, respectively. Phosphate complexes with calcium, therefore intake with milk should be avoided.

In adults, a small (n=16) uncontrolled prospective study reported reduced symptom scores following conventional treatment in 87% of patients (136). A trial of conventional treatment is therefore justified in symptomatic adults (137), although in many cases, symptoms of fatigue, low back pain, osteoarthritis, enthesopathy and spinal ligament calcifications may dominate, which generally do not respond to conventional therapy, nor does hearing loss (13). Furthermore, conventional medical therapy is often unpleasant, burdensome (14), and costly, requires monitoring and may be associated with side effects such as gastrointestinal symptoms, arterial hypertension or nephrolithiasis/nephrocalcinosis. There is consensus however that insufficiency fractures or pseudofractures, planned surgical procedures are indications for medical therapy in adults, while treatment may be considered in case of raised (bone) ALP and/or bone pain (13).

Concomitant vitamin D deficiency should be corrected with conventional vitamin D supplements *e.g.* cholecalciferol or ergocalciferol (138). Supraphysiological doses of active vitamin D analogs may be required to correct secondary hyperparathyroidism (139), but excessive vitamin D doses should be avoided due to risks of hypercalciuria, kidney stone formation and (rarely) hypercalcemia. Conventional treatment regimens with phosphate and vitamin D analogs improve intestinal phosphate absorption and paradoxically stimulate further FGF23 increases. Phosphate may exacerbate renal phosphate wasting (140) while active vitamin D attenuates phosphaturia (141). Of note, monotherapy with active vitamin D or CYP24A1 inhibition alone ameliorate rickets in preclinical mouse models (41, 142, 143) and may reduce the risk of secondary and tertiary hyperparathyroidism, supporting the use of the lowest possible dose of phosphate and higher doses of active vitamin D supplements.

In children, an initial dose of elemental phosphate between 20-40 (or 60) mg/kg body weight (0.7-1.3, up to 2.0 mmol/kg initial dose) is suggested, given as frequently as possible *e.g.* 4-6 daily divided doses in children (13). Doses > 80 mg/kg are to be avoided (25). Gradual titration may be used in the first weeks to avoid sudden gastrointestinal upset (13). Less frequent dosing *e.g.* 3-4 times daily may be used for maintenance or to improve compliance in adolescents and adults. In children, initial doses of alfacalcidol of 30-50 ng/kg (or calcitriol 20-30 ng/kg bodyweight) are suggested, which can usually be given once daily (evening dosage has been suggested) (25). Other active vitamin D analogs are not available in Belgium. Lower doses per body weight are recommended in adults. In general daily doses range between 750-1000 to 1600 mg of elementary phosphorus in 2-4 divided doses, and 0.75-1.5 µg of alfacalcidol (or 0.5 – 0.75 µg of calcitriol) (13, 25). However, dosage needs should always be individualized, since some patients need much higher or lower doses. With conventional therapy, phosphate dosages are not given with a goal of normalizing phosphatemia (keeping it at or above the lower limit is often not possible) but to improve growth and other symptoms and to normalize ALP, while avoiding secondary hyperparathyroidism and nephrocalcinosis (144). Active vitamin D analogs should be titrated to maintain PTH within the normal range but also to avoid hypercalcemia, hypercalciuria and kidney stone formation.

Although hypophosphatemia may exacerbate during pregnancy, the available evidence doesn’t clearly support a need for medical treatment in pregnant XLH mothers, since most will give birth uneventfully (145). Low phosphate levels in breast milk of XLH mothers has been reported in case reports, but adverse clinical outcomes have not been reported (146, 147). Similarly, no adverse effects of FGF23 excess on prenatal fetal-placental phosphate transport, breast milk phosphate concentrations or skeletal prenatal development are seen in mouse models (148, 149). Neonatal rickets is exceedingly rare, and there is currently insufficient evidence to formally recommend medical treatment for the sole indication of maternal or offspring skeletal health (18). Still, there is no international consensus on this topic (25). There is probably overuse of caesarean sections in this population, for no good reason (145).

#### Burosumab

In 2018, the European Medicines Agency (EMA) granted conditional market authorization to the fully humanized monoclonal anti-FGF23 antibody burosumab [formerly KRN23 (150)] for the treatment of XLH in children ≥ 1 year of age with a growing skeleton and radiographic evidence of bone disease (26). Late 2020, authorization was expanded for older adolescents and adults with radiographic evidence of bone disease, regardless of growth status.

In an open-label phase 2 trial, 52 children aged 5-12 years with XLH were randomized to receive burosumab without other conventional therapy, at a dose titrated to their phosphate level, every 2 or 4 weeks. The more frequent dosing led to more stable plasma phosphate concentrations, but the primary endpoint of the radiographic Rickets Severity Score was reduced in both groups (151). Most patients normalized their phosphate levels and TmP/GFR, and ALP levels declined. Greater height improvements were seen with the two-week dosing interval. Physical ability ameliorated and pain decreased in both groups (151). Another phase 2 trial confirmed the favorable efficacy and safety profile of burosumab in children aged 1-4 years (152).

Importantly, in an active-controlled open-label phase 3 trial, 61 children aged 1-12 years were randomized to receive conventional therapy or burosumab. Significantly greater improvements in radiographic healing of rickets (primary endpoint), growth, ALP and other biochemistries, lower-extremity deformities and mobility were observed with burosumab (153). Some patient-reported outcomes were significantly improved e.g. pain interference and physical health scores at week 40, although not for other outcomes or later time points (154). Some hypersensitivity and injection site reactions were noted in all trials, but there were no differences in serious treatment-related adverse events. Unfortunately, clinical trial data in adolescents remain lacking, despite the importance of the pubertal growth spurt. Nevertheless, the revised EMA market authorization would support continued use in adolescents when clinically indicated.

These data show that burosumab has the potential of improving clinical outcomes beyond current standard therapy. Notably, since it reduces the underlying renal phosphate wasting, burosumab has not been associated with complications of conventional therapy like nephrocalcinosis or secondary/tertiary hyperparathyroidism. However, burosumab is considerably more expensive than conventional medical therapy, and long-term outcomes as well as cost-effectiveness analyses are pending. Burosumab gained reimbursement in Belgium as of January 1^st^, 2021. Cost-sensitive criteria for use of burosumab in children in Belgium, proposed by the authors and approved by the competent authorities, are detailed in **Table 3**. Of note, these criteria still reflect the earlier EMA-approved indication (restricted to children with growing skeletons) and not the most recent version.

**Table 3:** Reimbursement criteria for burosumab in children in Belgium as of January 2021.

**Starting criteria**	• **Demographic criteria:** Children 1 year of age and older and adolescents with growing skeletons • **Diagnostic criteria:** With a diagnosis of X-linked hypophosphataemia o **Radiographic evidence of bone disease (rickets severity score ≥ 2)** o **Biochemical criteria:** ◾ Persistently low plasma phosphate (based on age-adjusted reference values) AND raised ALP (based on reference values for age) o And confirmed by either **genetic (or biochemical) criteria:** ◾ **genetic diagnosis** with *PHEX* mutation or appropriate family linkage ◾ Or, in case of no identifiable genetic mutation, raised serum **FGF23** concentration (> 30 pg/ml by Kainos assay, after discontinuation of conventional therapy for at least two weeks) • Following exclusion of all other causes of hypophosphatemia • **With at least one severe clinical symptom likely to improve with burosumab** o Lower limb bone deformity (genu varum or genu valgum) o Growth delay (≤ 20^th^ percentile for age and gender, according to national normative growth curves) o Dental abscesses during the past year o Chronic bone or muscle pain or joint stiffness o Reduced mobility (delayed gross motor development, need for walking aids, abnormal gait) o Presence of craniosynostosis • **Refractory to prior conventional medical therapy for at least 6 months, with complications of conventional medical therapy, or in case of intolerance for conventional therapy** • **Without renal insufficiency** *i.e.* estimated glomerular filtration rate > 30 ml/min/1.73 m² • **Physician criterion:** when prescribed at a university hospital by a pediatric nephrologist or endocrinologist, experienced in the treatment of XLH and participating in the European XLH registry
**Continuation criteria**	• **Reevaluation every year in children 1-12** years and **every 6 months in children ≥13 years; must meet all criteria below for continuation** • **Biochemical criteria:** o decrease in ALP compared to the initiation of treatment, AND o increase of plasma phosphate level compared to treatment initiation to levels above the lower limit of normal for age, OR ≥ 30% increase; OR increased renal tubular phosphate reabsorption to level > 0.84 mmol/L or ≥ 30% increase • **Radiographic criterion:** o Absolute decrease of the Rickets Severity Score of at least 1 point • **Improvement of at least one clinical symptom:** o Increased height Z-score o Improvement in bone deformity (genu varum or genu valgum) o Improvement in bone pain, joint stiffness or walking ability • **Evidence of continued growth (or potential):** height gain of ≥ 2 cm in the last year, or radiographically open epiphyses • **Compliance with clinical follow-up** at least every six months

The EMA-approved dose in children is a 2-weekly s.c. injection starting 0.8 mg/kg bodyweight, increased with 0.4 mg/kg dose increments (max. 2.0 mg/kg, cap at 90 mg dose) to achieve fasting plasma phosphate concentrations in the low-normal range for age. The average dose at this interval was 1.0 mg/kg in pediatric trials (25). Conventional treatment should be discontinued. Monitoring of peak phosphate levels at day 7-11 after injection, or before dosing after a three month period to achieve steady state is also suggested (25). In case of hyperphosphatemia, dosing should be withheld. The dose may need to be adjusted over time, but intervals for dose adjustment of 1-2 months are suggested (25). Creatinine levels should also be monitored, and treatment avoided in patients with incident renal insufficiency due to the theoretical risk of hyperphosphatemia and ectopic mineralization.

In adults, a phase 3 randomized trial (n=134) has compared placebo to a fixed 1 mg/kg bodyweight burosumab dose every four weeks. This normalized phosphatemia in almost 90% of patients. At 24 weeks, a significant decrease in joint stiffness and healing of active fractures was reported, with a safety profile similar to placebo (155). During the 24-week extension phase, all participants received open-label burosumab. Pseudofracture healing was confirmed in the group that switched from placebo to burosumab, and stiffness, pain and physical functioning and performance on the six-minute walking test improved significantly compared to baseline (156). Bone biopsies showed significant improvement in all osteomalacia indices (157). Thus, if approval is granted for use in adults by EMA (as has been done by its U.S. FDA counterpart, with a maximal dose of 90 mg), burosumab could represent an interesting treatment option for adult XLH patients suffering persistent bone and/or joint pain and disability, particularly from (pseudo-)fractures, despite a trial of optimal conventional therapy (25). Monitoring recommendations would be similar as in children (25).

#### Adjunctive Medical Therapies

Growth hormone is an off-label, yet theoretically attractive adjunctive therapy for XLH because it (transiently) increases phosphatemia and 1,25(OH)_2_D, lowers PTH and increases TmP/GFR, may improve height Z-scores without influencing body disproportion (158, 159) and might improve muscle strength. However, it doesn’t improve the underlying rickets and may increase ALP and exacerbate skeletal deformities (160). Moreover, it did not significantly improve adult height in long-term follow-up of a randomized controlled open-label study receiving conventional therapy (161). Clinical studies show that growth hormone therapy is more effective in prepubertal than in pubertal children (162). Although final height may be compromised despite conventional therapy in up to 60% of patients (163), extremely short children (Z-score ≤ -2.5) may be more likely to benefit (13, 164). Optimal control of rickets, PTH and ALP should be achieved before growth hormone therapy is considered in children with XLH (25). In Belgium, growth hormone therapy is not reimbursed for short stature related to XLH, although it has been obtained *via* medical need programs in the past (now no longer available).

Calcimimetics such as cinacalcet have been used to control secondary and tertiary hyperparathyroidism in XLH (165, 166). However, since they have been associated with severe side effects including hypocalcemia and QT-interval prolongation, their use should be limited (25).

Thiazide diuretics reduce hypercalciuria and may halt the progression of nephrocalcinosis in XLH (167). Thiazides should therefore be considered in case of arterial hypertension. However, adverse effects are common and require surveillance including hypotension, hypokalemia, hyponatremia, hypomagnesemia, hyperuricemia and increased insulin resistance. In contrast to hydrochlorothiazide, dipyridamole, which may reduce intrinsic renal phosphate leakage in some conditions, appears ineffective in XLH (168). The use of potassium citrate is not advised in XLH, because alkalinization may increase urinary phosphate precipitation (25).

Recently, a preclinical study in growing *Hyp* mice (the mouse model of *Phex* mutations) found that sclerostin inhibition increased phosphate and reduced FGF23 levels (169). Further evaluation of the efficacy and safety anti-sclerostin antibodies in the context of clinical trials is required. Other therapeutics targeting the FGF23 pathway *e.g.* suppressing the upregulation of osteopontin (170, 171) are also under development. Inadvertent treatment with bisphosphonates or other osteoporosis drugs, *e.g.* in case of misdiagnosis in patients with fractures, may induce deterioration and adverse skeletal effects (172).

#### Surgical Management

It is difficult to make firm evidence-based recommendations for orthopedic management in XLH (25). We recommend that care should be coordinated by an experienced orthopedic surgeon specialized in pediatric metabolic bone diseases. Insoles are not useful for pes planus in patients with varus or valgus knee deformity (25). In general, persistent or progressive severe deformity or disability despite ongoing optimal medical therapy may be an indication for surgery (25). Guided growth techniques by hemi-epiphysiodesis can be considered early as an alternative to more invasive options such as osteotomy (173), intramedullary nailing, external circular frames *e.g.* Ilizarov or combined techniques (174–177). Bed rest should be avoided as much as possible, and in those patients requiring it, careful monitoring for hypercalciuria is needed, which may warrant lowering of active vitamin D doses (25). Notably, medical treatment for at least 12 months in children and three to six months in adults, has been recommended before elective surgery, including dental implants (13, 25). Following surgery, prescribing rehabilitation is strongly recommended.

Repeated craniotomies may be necessary in symptomatic intracranial hypertension due to craniosynostosis (178). Neurosurgery may also be required for symptomatic Chiari malformation type 1, in which the cerebellar tonsils herniate through the foramen magnum and may compress the lower brainstem, upper spinal cord and/or cause syringomyelia (25). Up to 59% of children showed complete or partial sagittal suture fusion and 25% showed Chiari malformation type 1 in a recent large retrospective cohort study, however, most patients were asymptomatic (179). Rarely, symptomatic spinal enthesopathy or ossification of the posterior longitudinal spinal ligament may requiring laminoplasty, laminectomy or posterior decompression surgery (180–183).

Tertiary hyperparathyroidism with consequent hypercalcemia is a complication of longstanding high-dose phosphate supplements (184), although it has been noted in untreated patients too (18). It may be improved by partial or (sub)total parathyroidectomy, with or without ectopic parathyroid reimplantation. Clinicians should be aware that parathyroidectomy in these circumstances is usually followed by severe hungry bone syndrome with symptomatic hypocalcemia, which may require high doses of intravenous and oral calcium in combination with active vitamin D supplements (43, 185).

#### Monitoring

In patients receiving medical therapy, monitoring and adjustment of treatment doses should be based on measurements of calcium and phosphate in plasma and urine, creatinine, ALP and PTH at every visit (25). Notably, ALP may transiently increase during healing of rickets or signal the presence of pseudofractures. Increased ALP in otherwise well-controlled hypophosphatemia may signify poor compliance *e.g.* when patients improve their compliance shortly before clinic visits. If secondary hyperparathyroidism is present, alfacalcidol may be increased, phosphate doses decreased, or concomitant vitamin D deficiency may require replenishment.

Renal ultrasound to detect nephrocalcinosis or nephrolithiasis is recommended in medically treated XLH patients after one year and then every 1-2 years. Improvement of radiological signs of rickets can be seen after one year of treatment.

Patients receiving burosumab therapy may develop anti-drug antibodies, which may be accompanied by declining plasma phosphate levels and may require dosing increases when associated with clinical deterioration. Measuring anti-drug antibodies is not of clinical interest. The international guidelines suggest to measure 1,25(OH)_2_D every 3 to 6 months as a safety outcome in patients receiving burosumab therapy, together with monitoring for hypercalciuria (25). In patients receiving active vitamin D analogs and phosphate however, monitoring of 1,25(OH)_2_D is not recommended, because supraphysiological doses may be required to maintain PTH and calciuria within the desired range. Measuring FGF23 is not useful during follow-up of XLH patients, especially in patients treated with burosumab which may cause analytical interference (95).

## Discussion and Conclusions

This consensus document provides a broad, detailed and practical overview of clinical aspects of diagnosis and management of XLH, which can guide specialists in Belgium. Key findings and policy recommendations are summarized in **Table 4**.

**Table 4:** Summary of key policy recommendations.

Area	Findings
Epidemiology	• The estimated incidence of XLH in 1:20.000 or less live births, translates to less than six cases in newborns per year in Belgium, with a prevalence of less than 97 and 475 cases in the pediatric and adult population, respectively. • There remains a large gap in XLH diagnosis, treatment and follow-up in Belgium.
Diagnosis	• XLH has a broad differential-diagnosis. A correct diagnosis relies on the integration of clinical, radiological, biochemical and genetic findings. • We recommend a multimodal work-up of suspected XLH by an experienced clinician to exclude other diseases. • Pre-analytical and analytical challenges in the interpretation of plasma phosphate, alkaline phosphatase (ALP), phosphaturia, calciuria, 1,25-dihydroxyvitamin D and FGF23 should be taken into account.
Multidiscipinary care and follow-up	• We recommend referral to and follow-up by specialized multidisciplinary metabolic bone disease teams as well as protocols for transitional care between pediatric and adult specialists, and family-based outpatient clinics with pediatric-adult collaboration whenever possible. • We encourage caregivers to provide patients with information about the XLH patient association in Belgium.
Treatment	• Early medical treatment in children is advised to achieve optimal height, reduce skeletal deformities and reduce or avoid the need for surgery, to reduce musculoskeletal pain and to reduce dental complications. • Conventional medical therapy is often unpleasant, burdensome, and requires frequent monitoring and may be associated with side effects such as gastrointestinal symptoms, arterial hypertension or nephrolithiasis/nephrocalcinosis. • In a randomized controlled trial, burosumab resulted in significantly greater improvements in radiographic healing of rickets, growth and ALP, due to superior improvements in phosphatemia, TmP/GFR and 1,25-dihydroxyvitamin D compared to conventional medical treatment. • There is consensus that insufficiency fractures or pseudofractures, planned surgical procedures are indications for medical therapy in adults, while treatment may be considered in case of raised (bone) ALP and/or bone pain. • Given recent European approval of burosumab for XLH in adults, it could represent an interesting treatment option for patients suffering persistent bone and/or joint pain and disability, particularly from (pseudo-)fractures, despite a trial of optimal conventional therapy. • We recommend that orthopedic care should be coordinated by an experienced surgeon specialized in rare metabolic bone diseases.

Several key questions regarding XLH and other rare metabolic bone diseases remain unanswered (186): What are the best way to manage fatigue? What is the mechanism of pain? How can psychosocial support for patients and their families best be organized? How do rare metabolic bone diseases progress in ageing? Why do people with the same genetic mutation have different symptoms?

We recognize several limitations of these consensus recommendations. Most recommendations presented here, are based on expert opinion. We did not perform a systematic review nor used a formal GRADE or Delphi approach. However, recent international guidelines fulfill this need (25), and our recommendations can be considered a national translation of those guidelines. We believe national recommendations are useful, because Belgian policymakers have only recently initiated efforts to improve the care for patients with rare diseases, and no formal centers of expertise are officially recognized (currently, this task is delegated to all university hospitals). An important limitation is that not all specialties are represented among the authors. Specifically, a dentist or maxillofacial surgeon was not currently involved in XLH care pathways, highlighting the need to further liaise with other specialties in the care of XLH patients in our country. The Belgian burosumab reimbursement criteria as outlined in **Table 3** are quite strict and not necessarily evidence-based, but they represent a consensus between several of the authors, within the strict Belgian reimbursement context. In several neighboring countries, burosumab is reimbursed when prescribed for children or adults treated in centers of excellence. Ideally, this would also preferable in Belgium. An alternative would be to use the existing framework of so-called “college of expert physicians” to peer-review reimbursement requests. Moreover, we chose not to present separate guidelines for children and adults, since we believe in an integrated, life course approach. Nevertheless, some recommendations are not relevant for either children or adults. Finally, several authors have a conflict of interest, although there was no sponsor involvement in the development, writing or publication of these guidelines. The authors would also like to emphasize that these recommendations are not intended as a substitute for expert clinical judgement.

## Author Contributions

ML, JS, NG, EB, CH, JaL, KH, EL, and JV contributed to the conception of this work. ML, JS, EL, and JV wrote the first draft. All authors contributed to the article and approved the submitted version.

## Conflict of Interest

ML has received lecture and consultancy fees from Alexion, Amgen, Kyowa Kirin, Menarini, Sandoz, Takeda, UCB and Will-Pharma. JS has received lecture, consultancy fees, and conference support from Kyowa Kirin, Alexion, Eli-Lily, Ferring, Ipsen, Menarini, Novo Nordisk, Pfizer, Sandoz, and Siemens Healthcare. DT has received conference support from Novo Nordisk. NG, JLa, and KH have received consultancy fees from Kyowa Kirin. EB has received conference support from Novo Nordisk and Pfizer. CH has received conference support from Novo Nordisk and Ferring. EC has received consultancy fees from bioMérieux, Diasorin, Fujirebio, IDS, and Menarini. PH is an employee of GlaxoSmithKline but participates in his own capacity. J-FK has received consultancy fees and conference support from Heel Belgium, Sanofi, and TRB Chemedica. KW has received conference support from Alexion, Ferring, Kyowa Kirin and Novo Nordisk. CV has received conference support from Boehringer Ingelheim. GM has received consultancy fees from Alexion, Biomarin, Kyowa Kirin, and Pfizer. EL has received consultancy fees and travel support from Kyowa Kirin, Chiesi, and Recordati. JV has received conference support and consultancy fees from Alexion, Bellco, Ferring, Medtronic, and Kyowa Kirin.

The remaining authors declare that the research was conducted in the absence of any commercial or financial relationships that could be construed as a potential conflict of interest.
