# Corrigendum: Consensus Recommendations for the Diagnosis and Management of X-Linked Hypophosphatemia in Belgium

**DOI:** 10.3389/fendo.2021.686401

**Published:** 2021-05-25

**Authors:** Michaël R. Laurent, Jean De Schepper, Dominique Trouet, Nathalie Godefroid, Emese Boros, Claudine Heinrichs, Bert Bravenboer, Brigitte Velkeniers, Johan Lammens, Pol Harvengt, Etienne Cavalier, Jean-François Kaux, Jacques Lombet, Kathleen De Waele, Charlotte Verroken, Koenraad van Hoeck, Geert R. Mortier, Elena Levtchenko, Johan Vande Walle

**Affiliations:** ^1^ Centre for Metabolic Bone Diseases, University Hospitals Leuven, Leuven, Belgium; ^2^ Division of Pediatric Endocrinology, KidZ Health Castle, University Hospital Brussels, Vrije Universiteit Brussel (VUB), Brussels, Belgium; ^3^ Department of Pediatric Endocrinology, University Hospital Ghent, Ghent, Belgium; ^4^ Department of Pediatric Nephrology, Antwerp University Hospital, Antwerp, Belgium; ^5^ Laboratory of Experimental Medicine and Pediatrics, University of Antwerp, Antwerp, Belgium; ^6^ Pediatric Nephrology, Cliniques Universitaires St. Luc (UCL), Brussels, Belgium; ^7^ Paediatric Endocrinology Unit, Hôpital Universitaire des Enfants Reine Fabiola, Université Libre de Bruxelles, Brussels, Belgium; ^8^ Department of Endocrinology, University Hospital Brussels, Vrije Universiteit Brussel (VUB), Brussels, Belgium; ^9^ Department of Orthopaedic Surgery and Department of Development and Regeneration, Prometheus LRD Division of Skeletal Tissue Engineering, KU Leuven - University Hospitals Leuven, Leuven, Belgium; ^10^ XLH Belgium, Belgian XLH patient association, Waterloo, Belgium; ^11^ Department of Clinical Chemistry, University Hospital Center of Liège, University of Liège, Liège, Belgium; ^12^ Physical Medicine, Rehabilitation and Sports Traumatology, University and University Hospital of Liège, Liège, Belgium; ^13^ Division of Nephrology, Department of Pediatrics, University Hospital Center of Liège, Liège, Belgium; ^14^ Unit for Osteoporosis and Metabolic Bone Diseases, Department of Endocrinology and Metabolism, Ghent University Hospital, Ghent, Belgium; ^15^ Department of Medical Genetics, Antwerp University Hospital and University of Antwerp, Antwerp, Belgium; ^16^ Department of Pediatrics/Pediatric Nephrology, University Hospitals Leuven, Leuven, Belgium; ^17^ Department of Pediatric Nephrology, University Hospital Ghent, Ghent, Belgium

**Keywords:** Burosumab, fibroblast growth factor 23, osteomalacia, rickets, vitamin D, X-linked hypophosphatemia

There is an error in the Conflict of Interest statement. The correct statement is “ML has received lecture and consultancy fees from Alexion, Amgen, Kyowa Kirin, Menarini, Sandoz, Takeda, UCB and Will-Pharma. JS has received lecture, consultancy fees, and conference support from Kyowa Kirin, Alexion, Eli-Lily, Ferring, Ipsen, Menarini, Novo Nordisk, Pfizer, Sandoz, and Siemens Healthcare. DT has received conference support from Novo Nordisk. NG, JLa, and KH have received consultancy fees from Kyowa Kirin. EB has received conference support from Novo Nordisk and Pfizer. CH has received conference support from Novo Nordisk and Ferring. EC has received consultancy fees from bioMérieux, Diasorin, Fujirebio, IDS, and Menarini. PH is an employee of GlaxoSmithKline but participates in his own capacity. J-FK has received consultancy fees and conference support from Heel Belgium, Sanofi, and TRB Chemedica. KW has received conference support from Alexion, Ferring, Kyowa Kirin and Novo Nordisk. CV has received conference support from Boehringer Ingelheim. GM has received consultancy fees from Alexion, Biomarin, Kyowa Kirin, and Pfizer. EL has received consultancy fees and travel support from Kyowa Kirin, Chiesi, and Recordati. JV has received conference support and consultancy fees from Alexion, Bellco, Ferring, Medtronic, and Kyowa Kirin.

The remaining authors declare that the research was conducted in the absence of any commercial or financial relationships that could be construed as a potential conflict of interest.

There should be a change to Treatment section. A 0.4 mg/kg bodyweight dose is mentioned, whereas the most recently approved dose is a 0.8 mg/kg bodyweight dose. In the subsection “Burosumab” in the fifth paragraph, the first sentence should read as follows: The EMA-approved dose in children is a 2-weekly s.c. injection starting 0.8 mg/kg bodyweight, increased with 0.4 mg/kg dose increments (max. 2.0 mg/kg, cap at 90 mg dose) to achieve fasting plasma phosphate concentrations in the low-normal range for age.

The authors apologize for this error and state that this does not change the scientific conclusions of the article in any way. The original article has been updated.

